# A study of case management challenge for black grain eumycetoma during the ongoing war in Sudan

**DOI:** 10.1002/ccr3.9438

**Published:** 2024-09-16

**Authors:** Emmanuel Edwar Siddig, Imadeldin E. Aradaib, Ayman Ahmed

**Affiliations:** ^1^ Faculty of Medical Laboratory Sciences University of Khartoum Khartoum Sudan; ^2^ Department of Medical Microbiology and Infectious Diseases ErasmusMC, University Medical Center Rotterdam Rotterdam The Netherlands; ^3^ Molecular Biology laboratory, Faculty of Veterinary Medicine University of Khartoum Khartoum Sudan; ^4^ Swiss Tropical and Public Health Institute Allschwil Switzerland; ^5^ Faculty of Science University of Basel Basel Switzerland; ^6^ Institute of Endemic Diseases University of Khartoum Khartoum Sudan

**Keywords:** adherence to treatment, eumycetoma, herbal remedies, itraconazole, multidisciplinary management

## Abstract

**Key Clinical Message:**

This case highlights the significant challenges in the diagnosis and management of eumycetoma, particularly in regions like Sudan, where socioeconomic factors and ongoing conflict severely impact patient care. Delayed diagnosis and inadequate access to effective treatment can lead to poor adherence to prescribed therapies, prompting patients to resort to unproven self‐treatment methods. Comprehensive, multidisciplinary approaches that include education, improved accessibility to care, and addressing the impact of social determinants on health are essential to enhance the management of mycetoma, reduce disability rates, and improve patient outcomes in underserved communities.

**Abstract:**

Mycetoma is a chronic and debilitating infectious disease characterized by localized swellings and granulomatous lesions. It primarily affects individuals in tropical and subtropical regions and is caused by certain fungi or bacteria. This case report outlines the presentation, diagnosis, and management of a 37‐year‐old male from central Sudan with black grain eumycetoma, a challenging condition. The patient presented with recurring painless swelling in his right foot, which progressed over 5 years to include sinuses discharging black grain‐like materials. Despite initial treatment with itraconazole and folic acid, the patient discontinued medication due to war‐induced hardships including financial and accessibility to treatment and healthcare guidance, resulting in resorting to none‐effective and potentially harmful herbal remedies. Multidisciplinary management involving dermatologists, infectious disease specialists, and pharmacists supported with community health workers for health education is essential for enforcing adherence to treatment and successful recovery.

## 1 INTRODUCTION

Mycetoma is a stigmatized neglected tropical disease that primarily affects individuals of low socioeconomic status.^1^, ^2^, ^3^, ^4^ Several species of bacteria (actinomycetoma) or fungi (eumycetoma) that are mainly prevalent in tropical and subtropical regions are involved in the development of the disease.^5^, ^6^, ^7^, ^8^ Clinically, distinguishing between bacterial and fungal mycetoma requires a combination of diagnostic tools such as Fine Needle Aspiration, histology, culture, and modern molecular‐based methods.^9^, ^10^, ^11^, ^12^, ^13^, ^14^, ^15^, ^16^ One of the main challenges in the prevention and control of mycetoma is reaching final accurate diagnosis and characterization of the causative agent to identify the most effective treatment.^17^ Particularly, in countries like Sudan that are endemic with several diseases with similar clinical manifestations and overlapping symptoms such as cutaneous larva migrans, cutaneous tuberculosis, cutaneous Schistosomiasis, Leishmaniosis, Onchocerciasis, and several arboviral diseases with dermal presentation.^4^, ^18^, ^19^, ^20^, ^21^, ^22^ Additionally, the lengthy duration of treatment is a major challenge for the management of mycetoma cases, with eumycetoma patients often exhibiting poor responses to antifungal agents.^17^

Mycetoma is a major public health issue in the tropical and subtropical zones including Sudan, and it mainly spread across what is called the mycetoma belt, which extend from the far east of Asia through Africa and South America.^23^ However, cases are increasingly reported outside these assumed boundaries, likely driven by globalization and climate change.^24^ Despite local limitations in the diagnosis, surveillance, and treatment of mycetoma in Sudan, the country reports the majority of annual cases of mycetoma worldwide.

In this report, we present a case of a patient with eumycetoma containing black grain who was also co‐infected with tuberculosis.

## 2 CASE HISTORY

**FIGURE 1:**
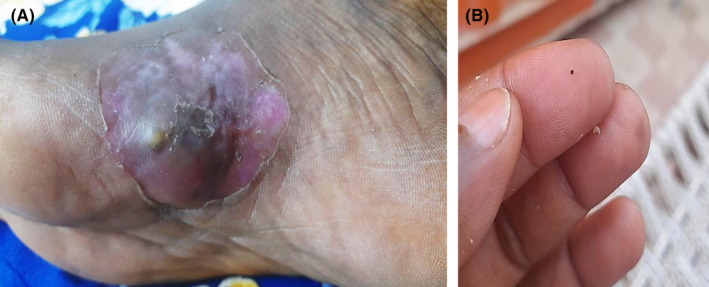
(A) Show the swelling at the medial aspect of the foot; (B) illustrating the discharged black grain.

**FIGURE 2:**
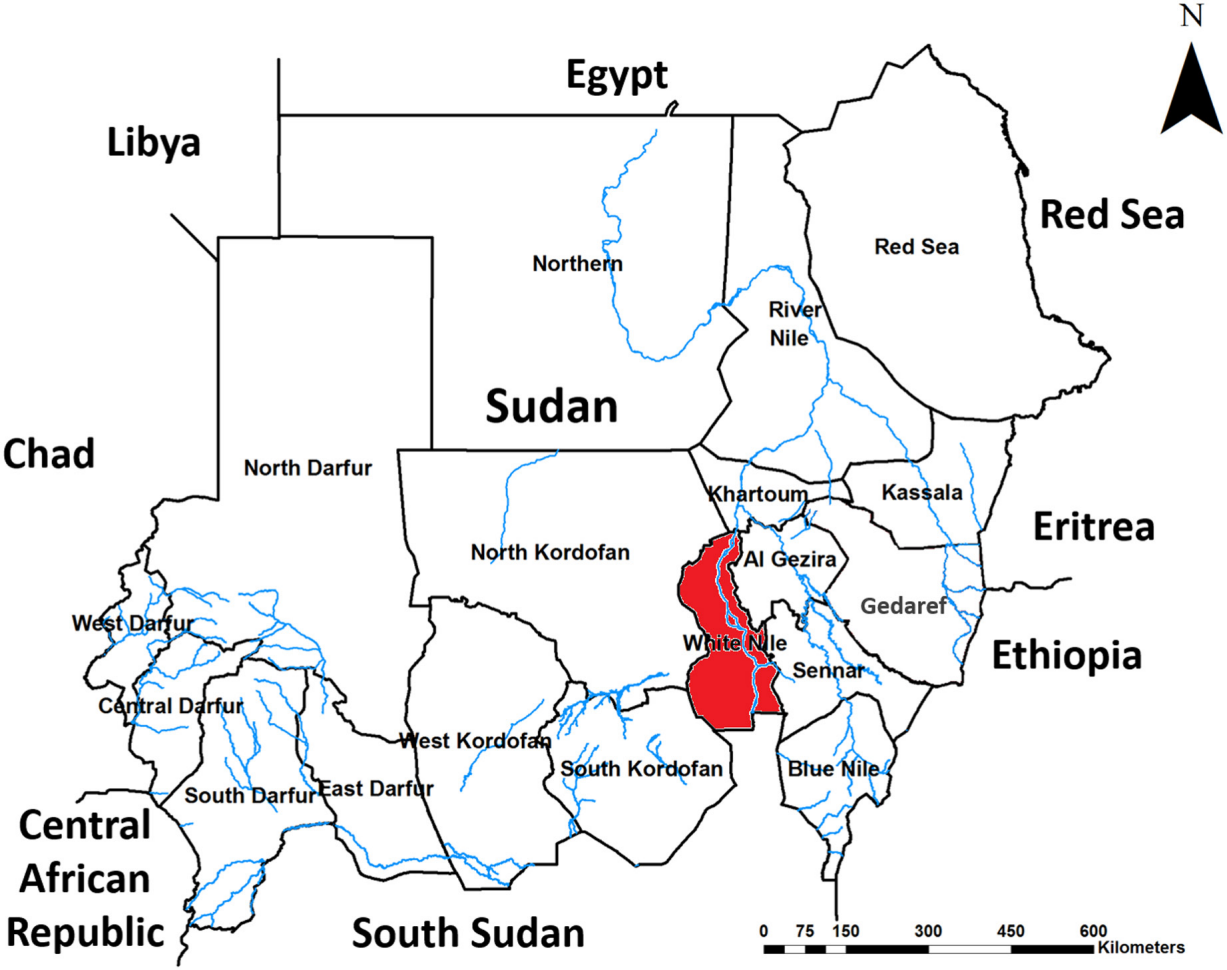
Map of Sudan indicates the original location of the case of black grain eumycetoma in Sudan; White Nile state highlighted in red.

A 37‐year‐old male freelancer from the White Nile State in central Sudan presented to the Surgical Department of Rufaa Teaching Hospital with a recurring, painless swelling in his right foot. The condition began 5 years ago with a small painless swelling on the medial side of his right foot, measuring 3.5 × 4 cm in diameter (Figure 1A). The infection progressed gradually, developing sinuses that discharged black grain‐like materials (Figure 1B). The patient mentioned having undergone surgical excision under local anesthesia previously, without any further treatment. He denied any history of local trauma or family members with similar conditions. Additionally, he reported a history of tuberculosis, for which he received medical treatment for it until successful treatment was confirmed a 2 years ago. The White Nile State is having open international borders with South Sudan, where he resides, is known to be endemic for various mycetoma infections (Figure 2).

## 3 METHODS

He had a normal pulse rate (78/min), respiratory rate (18/min), blood pressure (126/80), and temperature (37°C). Systemic examinations including cardiovascular (CVS), central nervous system (CNS), endocrine, and gastrointestinal (GIT) were all within the normal ranges. Local examination of the affected limb revealed a painless firm mass, non‐compressible and non‐ pulsatile, of less than 5 cm in size fixed to the deep structures and skin. A morphological examination of the skin showed that it was normal and no hypo or hyperpigmentation were noticed. There were multiple active and healed sinuses and discharge. Nonetheless, no regional lymphadenopathy was detected.

Examining the patient's liver functions showed serum bilirubin of 0.3 mg/dL, total protein of 7.8 g/d/L, serum albumin of 4.3 g/dL. Moreover, the tests revealed alkaline phosphatase of 93 U/L, aspartate aminotransferase (AST) of 19 U/L, and alanine aminotransferase (ALT) of 20 U/L. Testing renal functions of the patient, revealed normal blood urea of 27 mg/dL and serum creatinine of 0.41 mg/dL. His random blood glucose was 124 mg/dL. Since the patient was positive for tuberculosis a years before developing mycetoma a Viral screening for human immunodeficiency virus (HIV), Hepatitis B and C were requested and all were negative.

**FIGURE 3:**
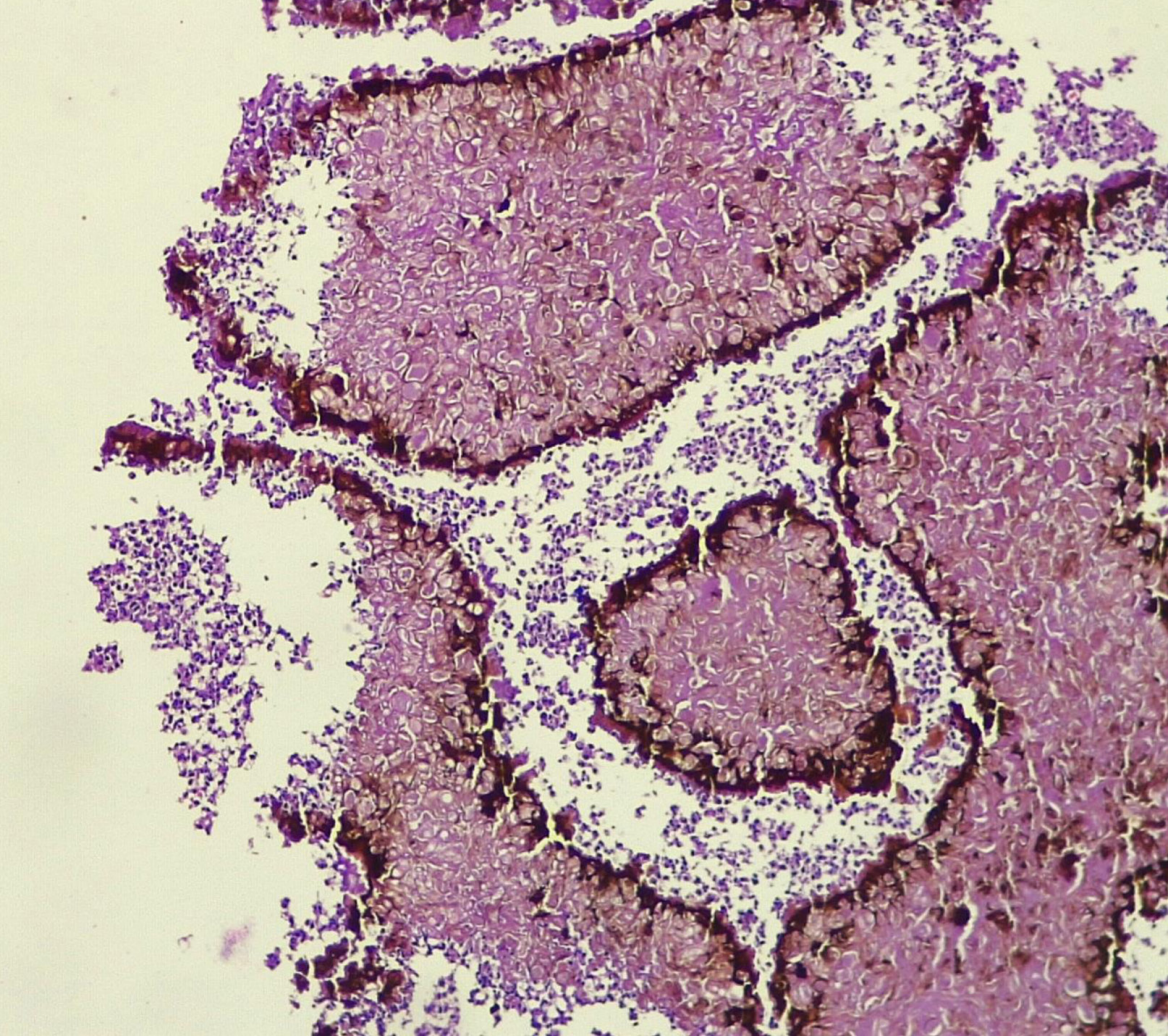
Illustrates the black grains of eumycetoma, which displayed a dark brown coloration at the edges and a lighter brown hue at the center. These grains were surrounded by inflammatory cells (H and E, X 40).

Histopathological investigations revealed the presence of a black‐grain surrounded by a zone of inflammatory cells (Figure 3). It also showed the existence of predominantly polymorphonuclear cells, lymphocytes, plasma cells, macrophages, and epitheloid cells.

## 4 CONCLUSION AND RESULT

**FIGURE 4:**
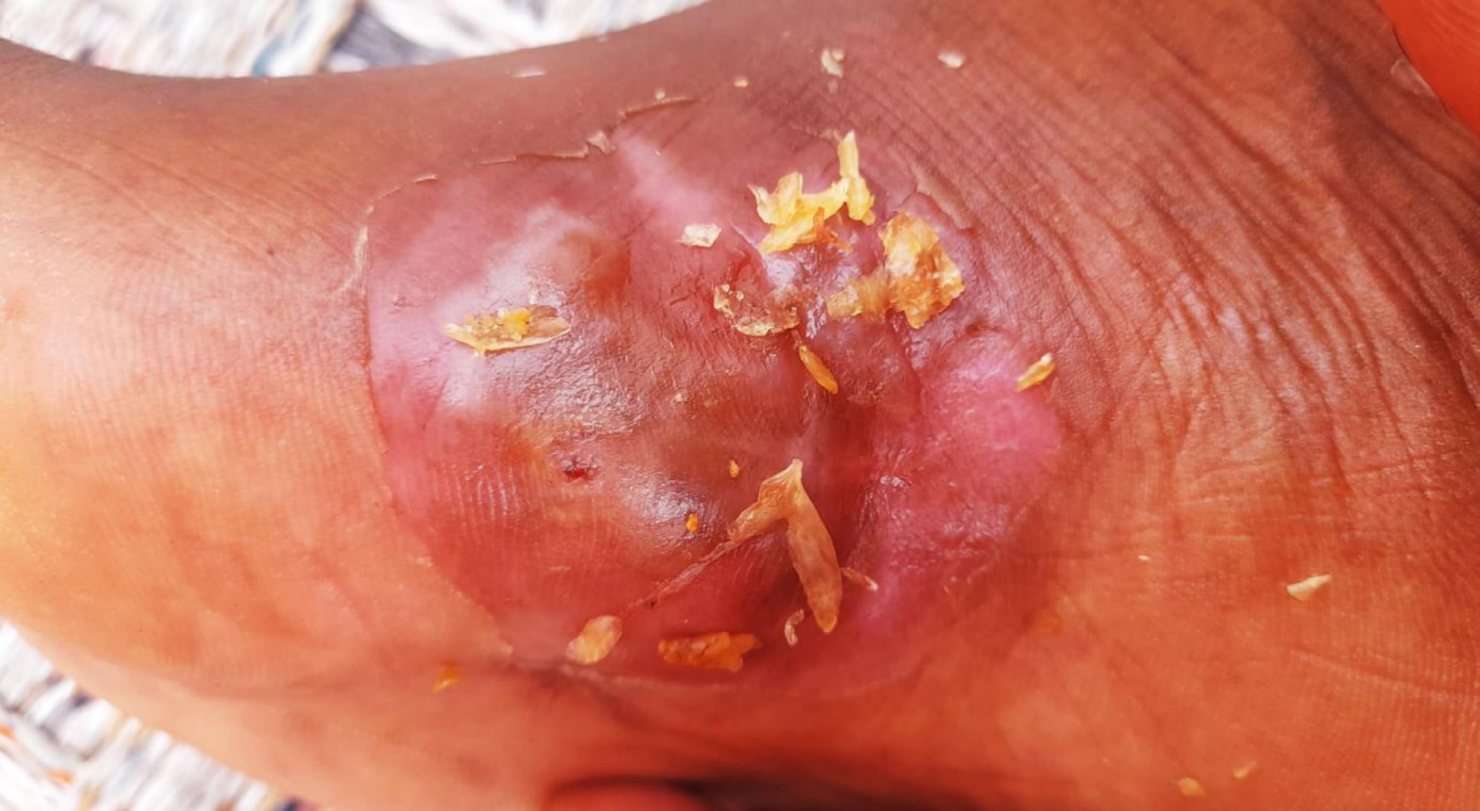
Shows the topical application of the garlic at the site of the infection.

After diagnosing the patient with black grain eumycetoma, the patient was administered 200 mg of itraconazole twice daily, as well as 5 mg of folic acid daily. The patient was advised to undergo clinical monitoring. However, after 3 months of starting the medical treatment, the patient discontinued the treatment and the virtual follow up visits due to the war complications including lack of resources, lack of network, insecurity stress, and shortage in drugs availability in the country. Instead, they began self‐treating with garlic (Figure 4), an herbal remedy they believed in.

As soon as connection with the patient was restored, we worked with them to address this issue. We facilitate accessibility to treatment. Education and advocacy were provided to help the patient understand the disease better and the importance of adhering to the prescribed medical therapy while avoiding invalidated herbal remedies because of the lack of information about their effectiveness and potential impacts on the infection site. The patient is currently under treatment and close follow up.

## 5 DISCUSSION

Here we presented a case of a 37‐year‐old male from the White Nile State in central Sudan with a history of recurrent painless swelling in his right foot, which started as a small swelling 5 years ago and progressed to develop sinuses discharging black grain‐like materials.^1^, ^2^, ^3^, ^23^ The patient had a past surgical excision without further prescribed treatment and he tested positive for tuberculosis, from which he was recently treated. The patient's vital signs were stable, and systemic examinations were unremarkable except for findings related to the foot swelling. Local examination revealed a painless firm mass on the affected limb with active and healed sinuses and discharge. However, no regional lymphadenopathy was noted. Laboratory investigations of liver and renal functions were all within normal limits and viral screenings for HIV and Hepatitis B, C, and E were all negative.^1^, ^25^, ^26^, ^27^, ^28^

A lesion deep surgical excisional biopsy was performed and histopathological examination revealed the presence of black grain surrounded by inflammatory cells, including polymorphonuclear cells, lymphocytes, plasma cells, macrophages, and epithelioid cells.^10^

The patient was diagnosed with black grain eumycetoma and prescribed itraconazole and folic acid, but discontinued treatment due to hardships related to the ongoing war,^29^, ^30^, ^31^ and accordingly he resorted to self‐treating with garlic. Efforts were made to improve access to treatment, educate the patient on the importance of medical therapy, and discourage the use of herbal remedies.

Unfortunately, along with the predominant poverty in Sudan, the country reports the heaviest burden of mycetoma worldwide.^32^ Despite this alarming statistic, Sudan is significantly lagging behind in patient advocacy and community engagement efforts, particularly in heavy burden regions. Sadly, the limited implemented advocacy is focused on Sennar state although the disease burden there is not as high as the White Nile, Gaziera, and Darfur states.^33^ This seems to be influenced by personal connections rather than strategic planning or systematic prioritization. This resulted in a wide spread ignorance among the community about the disease, associated risk factors, and effective personal measures for protection as well as awareness about the importance of seeking healthcare support as early as possible for a better case management and outcomes.^34^, ^35^, ^36^ This disparity in healthcare and public health services highlight a critical need for a national program that serves the country population regarding gender, age, ethnicity, or socioeconomic status. This will remove socioeconomic barriers and ensure equity in care. Considering that mycetoma is associated with high rate of disability mainly because of the delay in treatment that is commonly resulting in exposing the patient for inconsiderably invasive treatment through surgical amputation.^37^ Therefore, there is an urgent need for a nationwide initiative that expands the implementation of preventive and control measures including advocacy and educational services to the poor underserved communities in remote areas that are currently under significant health and socioeconomic burden of mycetoma or improve personal protection, early detection, and provision of effective treatment to everyone.

In this case, challenges faced include the limited awareness, treatment unavailability, and insecurity due to the ongoing war that have severely influenced the patient's adherence to medical treatment.^30^, ^31^, ^38^, ^39^ Multidisciplinary management involving dermatologists, infectious disease specialists, and health education and promotion professionals is critical for improving prevention, control, and case management of mycetoma case. Availability of effective and less invasive treatment (medications), long‐term monitoring and follow‐up are essential for successful treatment, prevent complications associated with eumycetoma, and eventually reduce the health and socioeconomic burden of the disease.

## AUTHOR CONTRIBUTIONS

**Emmanuel Edwar Siddig:** Conceptualization; data curation; formal analysis; investigation; methodology; supervision; validation; visualization; writing – original draft; writing – review and editing. **Imadeldin E. Aradaib:** Methodology; validation; visualization; writing – original draft; writing – review and editing. **Ayman Ahmed:** Conceptualization; data curation; formal analysis; investigation; methodology; supervision; validation; visualization; writing – original draft; writing – review and editing.

## FUNDING INFORMATION

None.

## CONFLICT OF INTEREST STATEMENT

The author reports no conflicts of interest in this work.

## CONSENT

Written informed consent was obtained from the patient to publish this report in accordance with the journal's patient consent policy.

## Data Availability

Data are available within the manuscript.
